# A novel inspection robot for nuclear station steam generator secondary side with self-localization

**DOI:** 10.1186/s40638-017-0078-y

**Published:** 2017-12-21

**Authors:** Jinke Li, Xinyu Wu, Tiantian Xu, Huiwen Guo, Jianquan Sun, Qingshi Gao

**Affiliations:** Guangdong Provincial Key Laboratory of Robotics and Intelligent System, Shenzhen Institutes of Advanced Technology, Chinese Academy of Sciences, 1068 Xueyuan Avenue, Shenzhen, 518055 China

**Keywords:** Nuclear station, Steam generator, Robotic system, Inspect, Localization

## Abstract

Nuclear energy is one of the most important clean energy on earth presently. The steam generator secondary side is the key device of nuclear power station. As an important branch of special robot, nuclear robot is the most convenient and effective mean to inspect the steam generator. This paper describes one robot system which could help users inspecting tubes and locating the robot inside the steam generator. The main part of this system is a climbing robot which can move inside the steam generator carrying a PT or telescopic arm. Four cameras are installed on the robot and PT to send real-time videos back for analysis. Experiments show that this system works stably and the localization is accuracy and effective.

## Background

As one of the most important branches of robotics, climbing robots have been studied well and extensively applied in various fields since the first climbing robot was designed by Nashi [1]. Climbing robots can be divided into categories according to how each stick on the wall. Magnetic adhesion was widely applied in climbing robots, and many robots have adopted this method [2]. In the early 1990s, a climbing robot that moves on the wall based on a vacuum chunk was built up by researchers from Japan and the USA [3, 4]. Gecko [5] is one new type of climbing robot that can move on many kinds of slippy surfaces by using Van der Waals force to stay on the wall. In recent years, climbing robots that can move on special materials such as cloth and trees were created. CLASH [6] is the first robot that could move on cloth, and Clothbot [7] is the first wheel robot for climbing on cloth. Treebot [8, 9] can climb on the tree with its two claws.

Many robots, including climbing robots, are designed for nuclear stations [10–17]. A steam generator is the key device in a nuclear power station in which heat is passed from the coolant in the primary circuit to the water in the secondary circuit to generate high-pressure and high-temperature steam for electricity. The steam generator is a huge cylindrical object with a 3 m diameter, and to enhance the efficiency of heat exchange, thousands of tubes are inside of it, making it a fragile device vulnerable to various problems. To make sure the steam generator works safely, it needs to be inspected annually. The key problem we need to solve is to get pictures of tubes which are inside of the steam generator secondary side in real time. The inner structure of the steam generator makes it impossible to check it manually, because the space is too small for person to fit. Obrutsky [18] summarized some technologies for steam generator tube inspection. So a robot with cameras which could move inside the steam generator secondary side is very convenient. It should be noted that broken tubes can be repaired conveniently. Therefore, the real-time positioning of the vehicle is meaningful.

In this paper, we develop an intelligent robotic system which includes a climbing robot with cameras which can transmit the videos of tubes in real time and it can move inside the steam generator secondary side and help us to analyze if the tubes are safe. Lastly, we propose an approach to locate the vehicle.

## System design

**Fig. 1:**
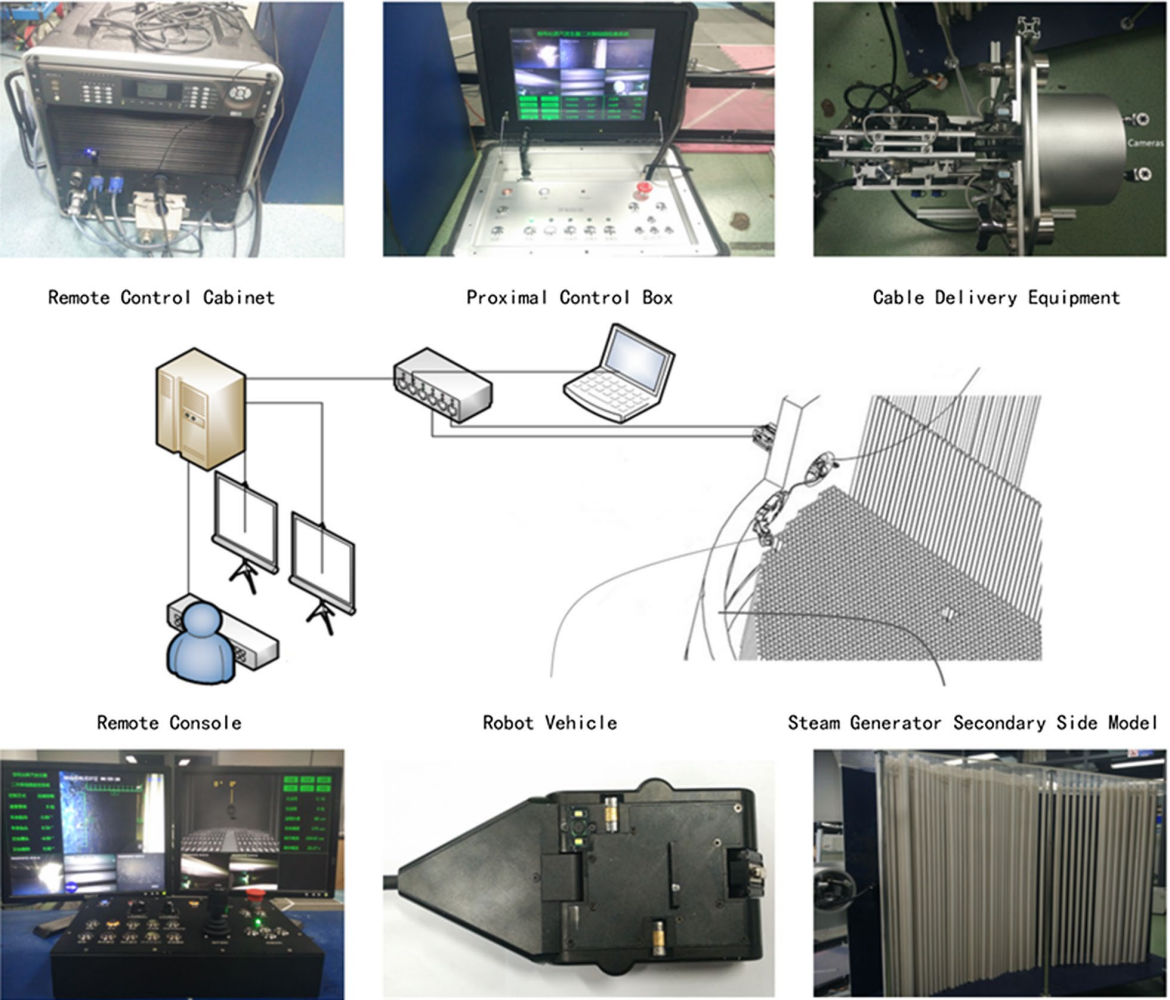
Schematic diagram of the whole system

The robot system used in the nuclear steam generator secondary side for inspection consists of seven components: robot vehicle, Pan-Tilt (PT) and telescopic arm, cable delivery equipment, remote control cabinet, remote console, proximal control box, and junction box. The connection among them is shown in Fig. 1.

Remote console plays a leading role when this system works normally as it is placed nearly 60 m away from the steam generator secondary side, but if the remote console is not working properly, the proximal control box would take over control of this system for safety.

### Robot vehicle

**Fig. 2:**
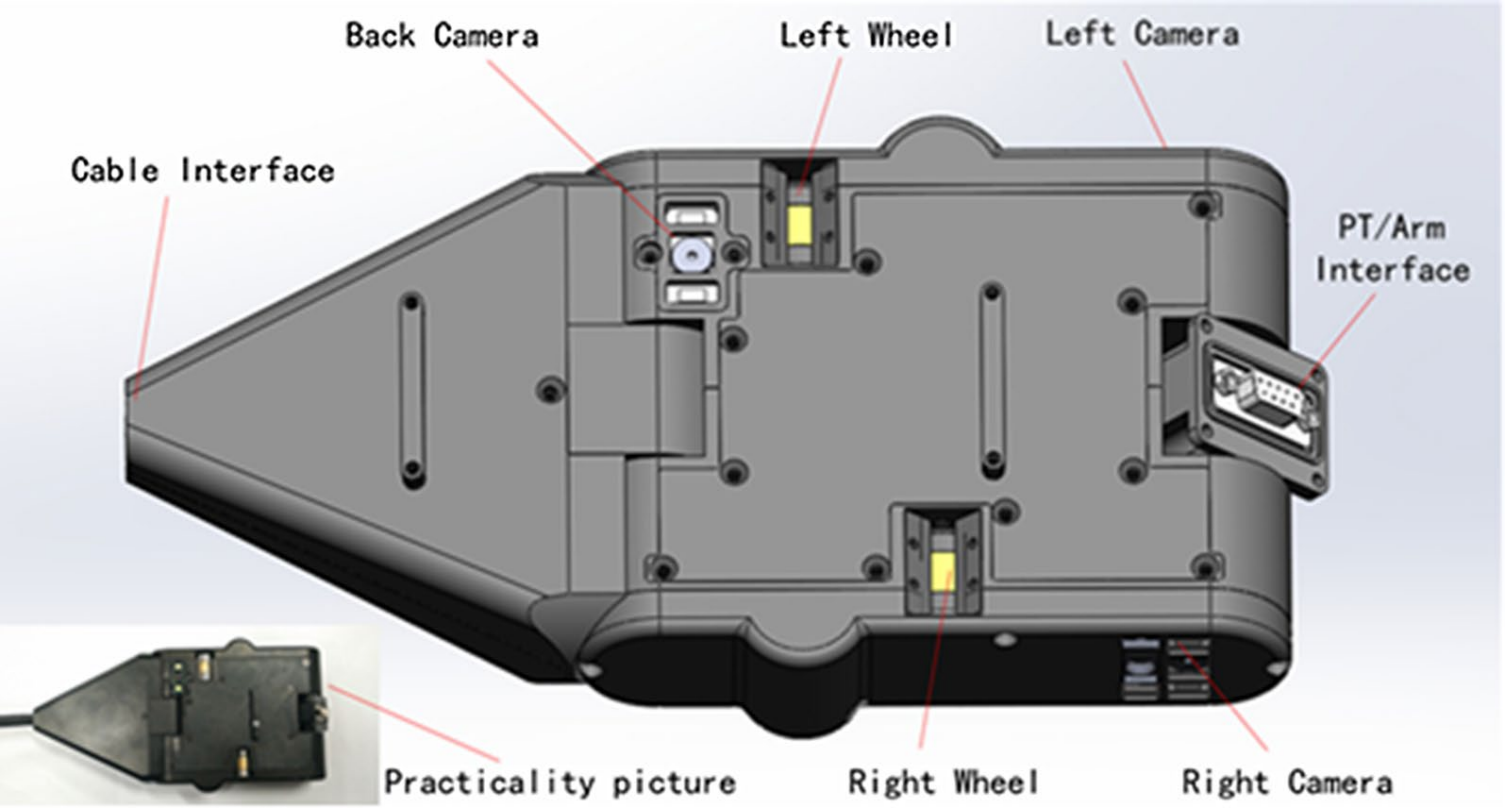
Robot vehicle

The main part placed in the steam generator is the robot vehicle. This vehicle can stick on the tube wall of the steam generator through magnetic adhesion and move in any direction according to the user’s input. The magnalium-made body is the main part of this vehicle, and two differential permanent magnet-made wheels are parallel to each other on the body. A servo motor is installed in front of this vehicle and used for raising the PT or telescopic arm. Three cameras are installed on the back, left side, and right side, respectively, and a LED lamp is put along each camera for filling light. Distance sensors are on the left and right sides of this vehicle (Fig. 2).

Safety is a priority in the nuclear industry, and we have made many preparations to avoid accidents when this vehicle is working in the steam generator. For example, the vehicle’s wheels are made of neodymium iron boron (NdFeb), a strong permanent magnet. These wheels will prevent the vehicle from dropping off even if all the devices are powered off. One cable is dragged in the tail of this vehicle and used for transferring videos and commands. We can even pull the vehicle out by the cable in extreme cases.

### PT and telescopic arm

Two different devices can be installed in front of the vehicle.

**Fig. 3:**
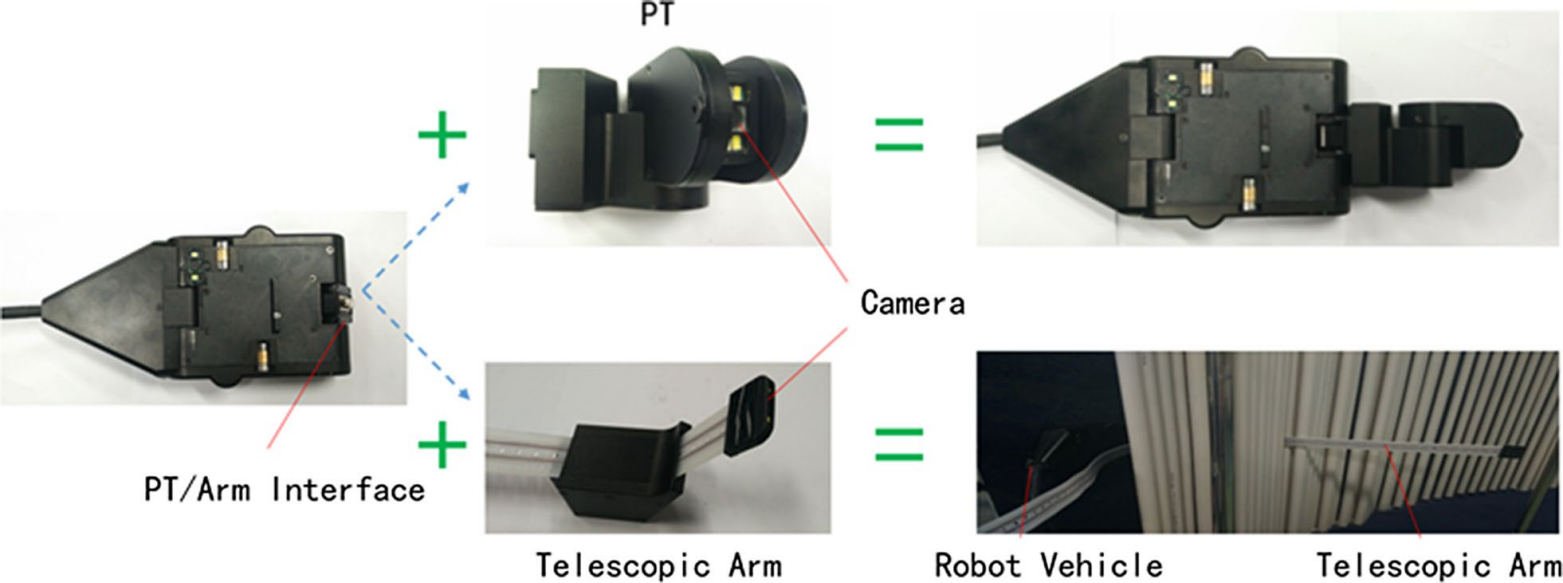
PT/telescopic arm and interface in front of the vehicle

One is the PT, which has two degrees of freedom to control a camera that is installed in front of it. The swinging head motor provides a range of $-\,90^{\circ }$ to $120^{\circ }$ and the pitch motor can provide a pitch angle from $-\,80^{\circ }$ to $80^{\circ }$. This device can check the tubes nearby of the tube wall and locate the camera precisely. We can control the position and gesture directly (Fig. 3).

Another device is the telescopic arm, which can extend 1.5 m for checking tubes in the center of the steam generator. Made of flexible material, the telescopic arm can be put in the tubes. The center of the telescopic arm contains many holes, so it could be fixed and moved by motor. A camera in front of the telescopic arm allows transfer of video for checking tubes and further analysis.

Installation of the PT or telescopic arm in front of the vehicle is convenient for users.

### Remote control cabinet

Remote control cabinet controls the main program of the robot system. It calculates the user’s command collected from the remote console or proximal control box and sends commands to the robot vehicle and cable delivery equipment. Each device in the cabinet has its special purpose. Of course, the main program runs on one small industrial personal computer (IPC). Since the PAL video signal from the robot vehicle cannot be used directly, a hardware driver is needed to convert the PAL signal into digital format for further use. Emergency energy can be used for more than an hour to prevent the remote system from shutting down in case of incidents such as power loss. We also build a power manager to manage the remote system’s power supply.

### Remote console

Remote console is the interaction of user and control system. It consists of many components such as buttons, potentiometers, and levers to enable multiple user control commands. Besides, two screens in the remote console make it convenient for users to monitor the videos inside the steam generator and the vehicle’s state.

One screen displays the four videos from the vehicle and PT or telescopic arm, and some important states of the system. These videos will help users to check tube damage and will be stored for further analysis. We noticed it is difficult for users to control the vehicle inside the steam generator based only on the four videos, since users know little about where the vehicle is and its gesture. Another screen, which shows a virtual 3D model of the steam generator and simulates the inside of the steam generator, is therefore necessary. The second screen also shows two videos from the cable delivery equipment to help users insert the robot vehicle.

### Proximal control box

The proximal control box includes one control panel, one screen, and one IPC. An independent program runs on the IPC. If the remote control cabinet works normally, the program nearby receives data only from the remote one. But if the remote control cabinet does not work, the program will take over control of the system.

The users would operate the nearby control panel and watch the nearby screen, which displays all six videos and some important states when the vehicle is put in. After the vehicle is put in, users would go to the control room and operate the remote console to avoid the radiation. In extreme cases, however, even if the remote control cabinet is power off, the proximal control box could be used for moving the vehicle out of the steam generator.

## Localization

### Establishing a coordinate

**Fig. 4:**
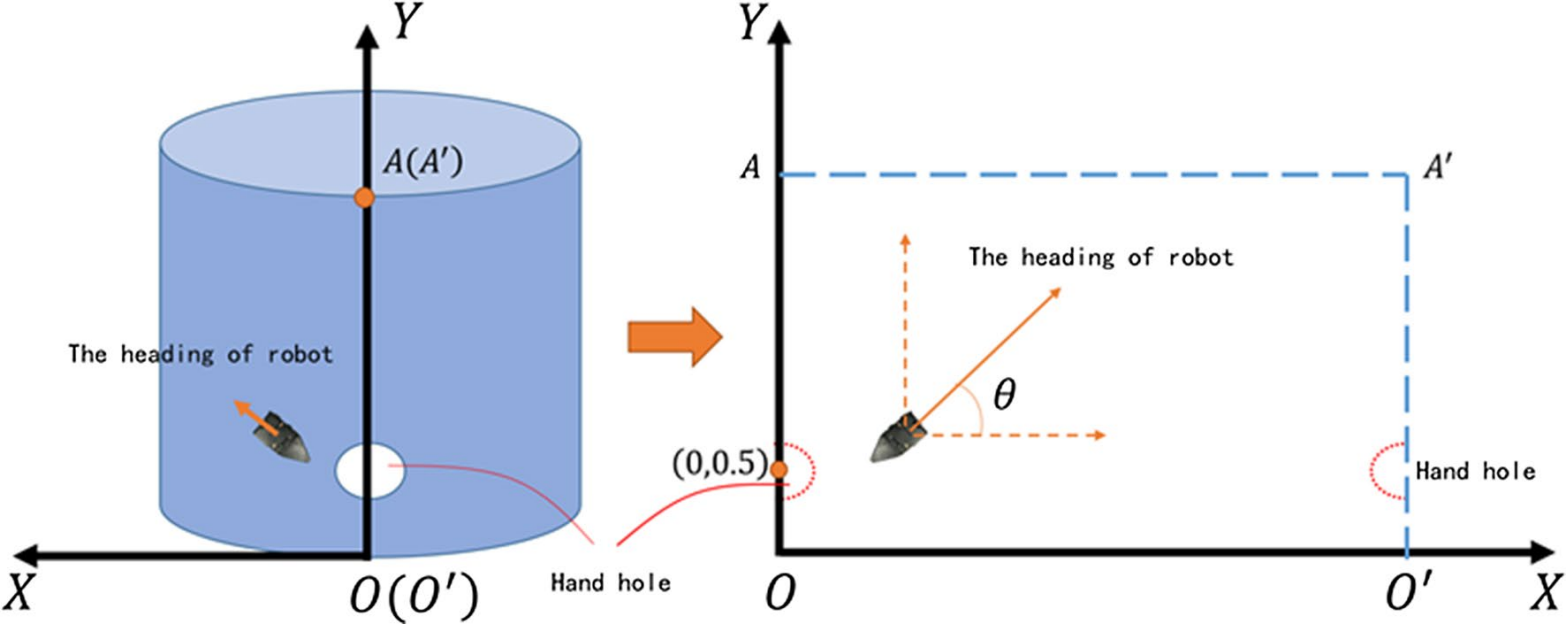
3D model of steam generator and coordinate of whole system

To locate where the robot is in the stream generator, we must establish a coordinate for prescribing the whole system, which includes the steam generator and the robot conveniently. In this paper, we use the Cartesian coordinates to describe this system.

As shown in Fig. 4, we establish a Cartesian coordinate for the environment and robot vehicle. There are a lot of tubes in the tube wall. The point we care about is the robot’s position on the tube wall, so we only need to consider how to simulate the tube wall model. To the right of Fig. 4, we describe how to establish a Cartesian coordinate with the model. The steam generator is a cylindrical object with two symmetrical holes for putting the robot into it. In fact, the hole’s height is 0.5 m, and we define the initial point we put the robot as (0, 0.5) according to which hole the robot goes in. The cylindrical object should be opened as a rectangle along with the line crossing the center of the robot and perpendicular to the ground. The point that the line intersects with the ground as zero point is then defined.

### Kinematics model

**Fig. 5:**
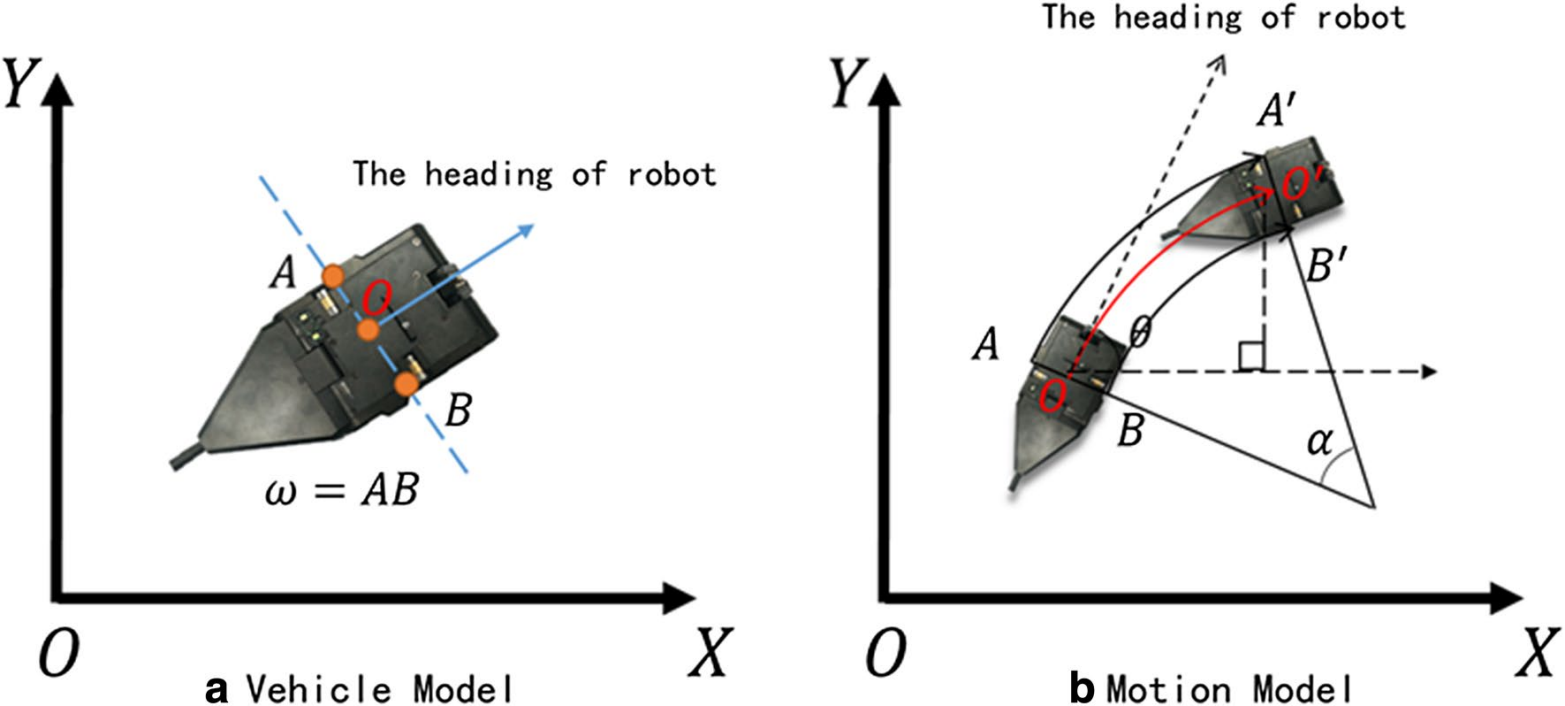
Mathematical model of robot in the coordinate system

Figure 4 shows the mathematical model of robot in the coordinate system. In Fig. 5, we establish the robot’s mathematic model. We simplify the two wheels of the robot in the same axis. Point *A* and point *B* represent the center of the left wheel and right wheel, and point *O* means the center of the whole robot, of which we research in this paper.

In the system we built, we now analyze kinematics model of the robot. In Fig. 5, we suppose that the current position of the robot is at point *O*, which is $(x_{0},y_{0})$. The angle between its head direction and *X* axis is $\theta$ . We first consider that the vector of the two wheels is same sign. The two wheels go an arc rather than straight line. We can know the left wheel’s displacement $S_{\mathrm{L}}$(arc$AA{}'$ ) and right wheel’s displacement $S_{\mathrm{R}}$(arc$BB{}'$ ). The position of the robot then moves to point $O{}'$, which is $(x_{1},y_{1})$ . We know the width of the vehicle $\omega (AB,A{}'B{}')$.

When $S_{\mathrm{L}}$ and $S_{\mathrm{R}}$ are very small, we can list the following equations:

1 $$\begin{aligned} \ \alpha =\frac{S_{\mathrm{L}}-S_{\mathrm{R}}}{\omega } \end{aligned}$$

If $S_{\mathrm{L}}\not \equiv S_{\mathrm{R}}$, we can list the following equations:

2 $$\begin{aligned} x_{1}& {}= x_{0}+2\left( \frac{S_{\mathrm{L}}\omega }{S_{\mathrm{L}}-S_{\mathrm{R}}}-\frac{\omega }{2}\right) \hbox {sin}\frac{\alpha }{2}\hbox {cos}\left( \theta -\frac{\alpha }{2}\right) \end{aligned}$$

3 $$\begin{aligned} y_{1}& {}= y_{0}+2\left( \frac{S_{\mathrm{L}}\omega }{S_{\mathrm{L}}-S_{\mathrm{R}}}-\frac{\omega }{2}\right) \hbox {sin}\frac{\alpha }{2}\hbox {sin}\left( \theta -\frac{\alpha }{2}\right) \end{aligned}$$

If the vector of two wheels are different, we can get the following equations:

4 $$\begin{aligned} x_{1} & {} = x_{0}+\left( \frac{S_{\mathrm{L}}\omega }{S_{\mathrm{L}}-S_{\mathrm{R}}}-\frac{\omega }{2}\right) \hbox {sin}\alpha \hbox {cos}(\theta -\alpha ) \end{aligned}$$

5 $$\begin{aligned} y_{1} & {}= y_{0}+\left( \frac{S_{\mathrm{L}}\omega }{S_{\mathrm{L}}-S_{\mathrm{R}}}-\frac{\omega }{2}\right) \hbox {sin}\alpha \hbox {sin}(\theta -\alpha ) \end{aligned}$$

If $$S_{\mathrm{L}}=S_{\mathrm{R}}$$:

6 $$\begin{aligned} x_{1} & {} = x_{0}+S_{\mathrm{L}}\hbox {cos}\theta \end{aligned}$$

7 $$\begin{aligned} y_{1} & {}= y_{0}+S_{\mathrm{L}}\hbox {sin}\theta \end{aligned}$$

Equations (1)–(7) are the state transition matrix in the ideal state of this system.

### Image model

Three cameras are on the robot vehicle. This paper uses images from these cameras, especially from the camera on back of this robot vehicle, to help for localization.

1. Hough transformation:
  Hough transformation is a convenient and effective method that accounts for the particularity of the application environment. In this paper, we achieved two consecutive frames from the camera. The many straight tubes, which are perpendicular to the ground in steam generation, enable the calculation of the robot vehicle’s head direction. Many lines should be detected, and averaging the slope of these lines would give the robot’s head direction more precision.
2. Template matching:
  Using a subimage 300*300 from the former frame image as a template, the template’s position is known as $p\left( x_{0},y_{0} \right)$. Then next frame image would then be rotated according to the difference between the former frame image’s result of Hough transformation $\theta _{0}$ and the next frame image’s result of hough transformation $\theta _{N}$. Detecting the template in the next frame image so we could get the new position $p'\left( x_{N},y_{N} \right)$. Rotating coordinate of the next image through $p'\left( x_{N},y_{N} \right)$ and the angle difference between the results of two images Hough transformation. The difference between these two coordinates is the incremental $\Delta \left( x,y \right)$ of this coordinate system:
  8 $$\begin{aligned} \Delta (x,y)=f(p{}'(x_{N},y_{N}),(\theta _{0}-\theta _{N}))-p(x_{0},y_{0}) \end{aligned}$$
  where *f* is the coordinate system rotation function.
3. Location model:
  After an incremental change $\Delta (x,y)$ in the image coordinate system, we can calculate the incremental change $\Delta {}' (x,y)$ in the world coordinate system. According to the position $p_{1}(x_{0},y_{0})$ and incremental change $\Delta {}' (x,y)$, we can then get the robot’s current position $p_{1}(x,y)$:
  9 $$\begin{aligned} \Delta ' (x,y)= & {} -\,a\Delta (x,y)\end{aligned}$$
  10 $$\begin{aligned} p_{1}(x,y)= & {} p_{1}(x_{0},y_{0})+\Delta ' (x,y) \end{aligned}$$
  where *a* is the conversion constant between pixels and mm.

### Particle filter

As we simplify the two wheels of robot in the same axis, there must be a little error. And the result of the image model is not Gaussian. So we look for some sensor fusion methods to improve the accuracy of localization. While the robot vehicle moves slowly, and the calculation is carried on the pc, so we have enough time and computing power to implement a more accurate algorithm such as particle filter. In this paper, we fuse odometer and image models through particle filter method. Below are the steps to follow.

1. Initialization:
  Build a set of particles $(x_{0}^{i})_{i=1}^{N}$, read data from the odometer and the acceleration, and calculate $P(x_{0})$ according to the odometer model. Then sample *N* particles $(i=1,2,\ldots ,N)$ from it and give each particle weight of 1 / *N*.
2. SIS:
  First, get observed value $z_{k}$ through image location model, sampling *N* particles $x_{k}^{i}(i=1,2,\ldots ,N)$ from function $q(x_{k}\mid x_{k-1},z_{k})=p(x_{k}\mid x_{k-1})$. Then, calculate the weight of each particle:
  11 $$\begin{aligned} \omega \left( x_{k}^{i}\right) =\omega \left( x_{k-1}^{i}\right) \frac{p\left( z_{k}\mid x_{k}^{i}\right) p\left( x_{k}^{i}\mid x_{k-1}^{i}\right) }{q\left( x_{k}^{i}\mid x_{k-1}^{i},z_{1:k}\right) } \end{aligned}$$
  At last, calculate the normalized weight of each particle: $w_{k}^{j}=w_{k}^{j}/\sum _{j=1}^{N}w_{k}^{j}$.
3. SIR:
  Judge if it needs to resample through effective sampling scale $N_{\mathrm{eff}}$ and the default threshold $N_{th}$ , if it does, replace small weight of particles with high weight of particles according to normalized weight $\tilde{\omega _{k}}(x_{0:k}^{i})$ from $(x_{k}^{i})_{i=1}^{N}$ . If it does not need to resample, continuing to the next step.
4. Output:
  Output a set of particles $\{x_{k}^{i},\omega _{k}^{j}\}_{i=1}^{N}$.
5. Refresh:
  Time $k=k+1$, read a new next frame image, calculate a new observed value, and return to step 2.

## Experiments

In this paper, we test the running state of the vehicle in many cases, such as the working state of water, the temperature changes in continuous work. Experiments show that the vehicle worked well in these cases, the vehicle responds quickly according to commands, and the control system is stable and safe.

This paper creates many experiments for positioning, and what we are concerned with is the positioning of the different movement processes.

**Fig. 6:**
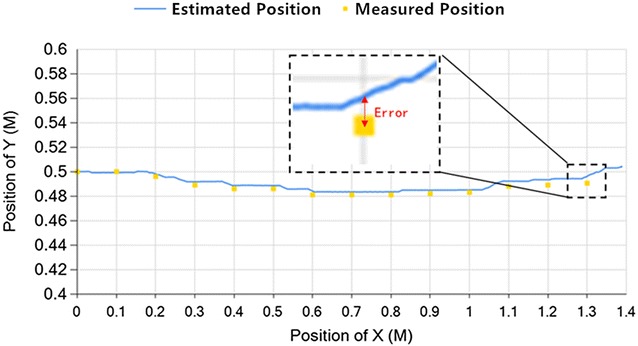
Long straight line

From Fig. 6, the yellow points present the points that the vehicle passed through actually, which are the height of its geometry center measured by a laser rangefinder on the datum lines of *X* axis. The blue line is the trajectory of the vehicle calculated by the localization algorithm. The error is considered as the difference between yellow point and the point at which the blue line intersects the datum line of *X* axis. The range of the vehicle’s regular activity is at a height about 1 m, and the width of the vehicle is 0.12 m. We consider the error that below half of the vehicle’s width is acceptable, which is 6%.

**Fig. 7:**
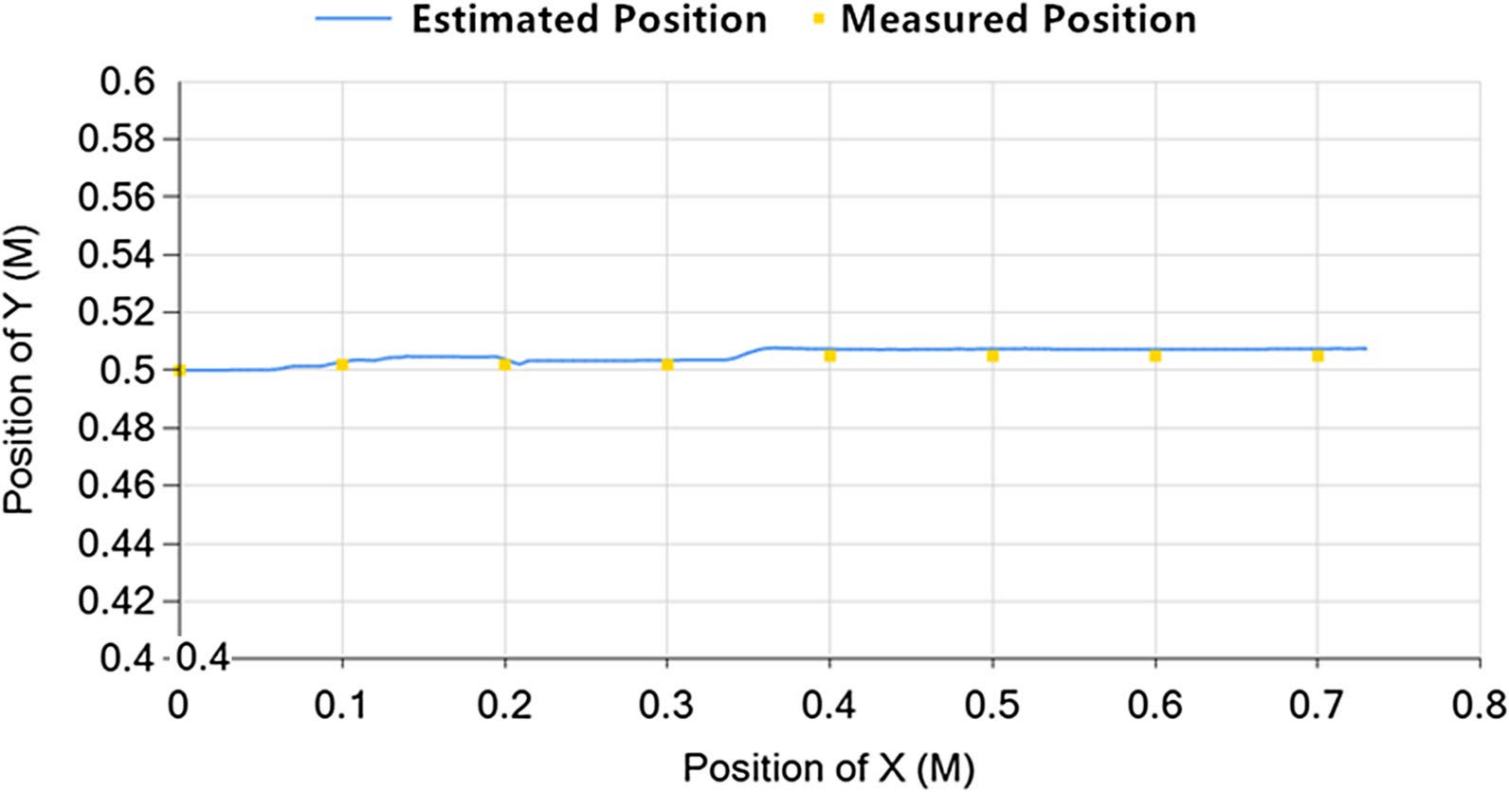
Short straight line

According to Figs. 6 and 7, if the vehicle is moving along a straight line, the location error is below 0.8%. So as it moves along a straight line in the steam generator, the localization is accurate.

**Fig. 8:**
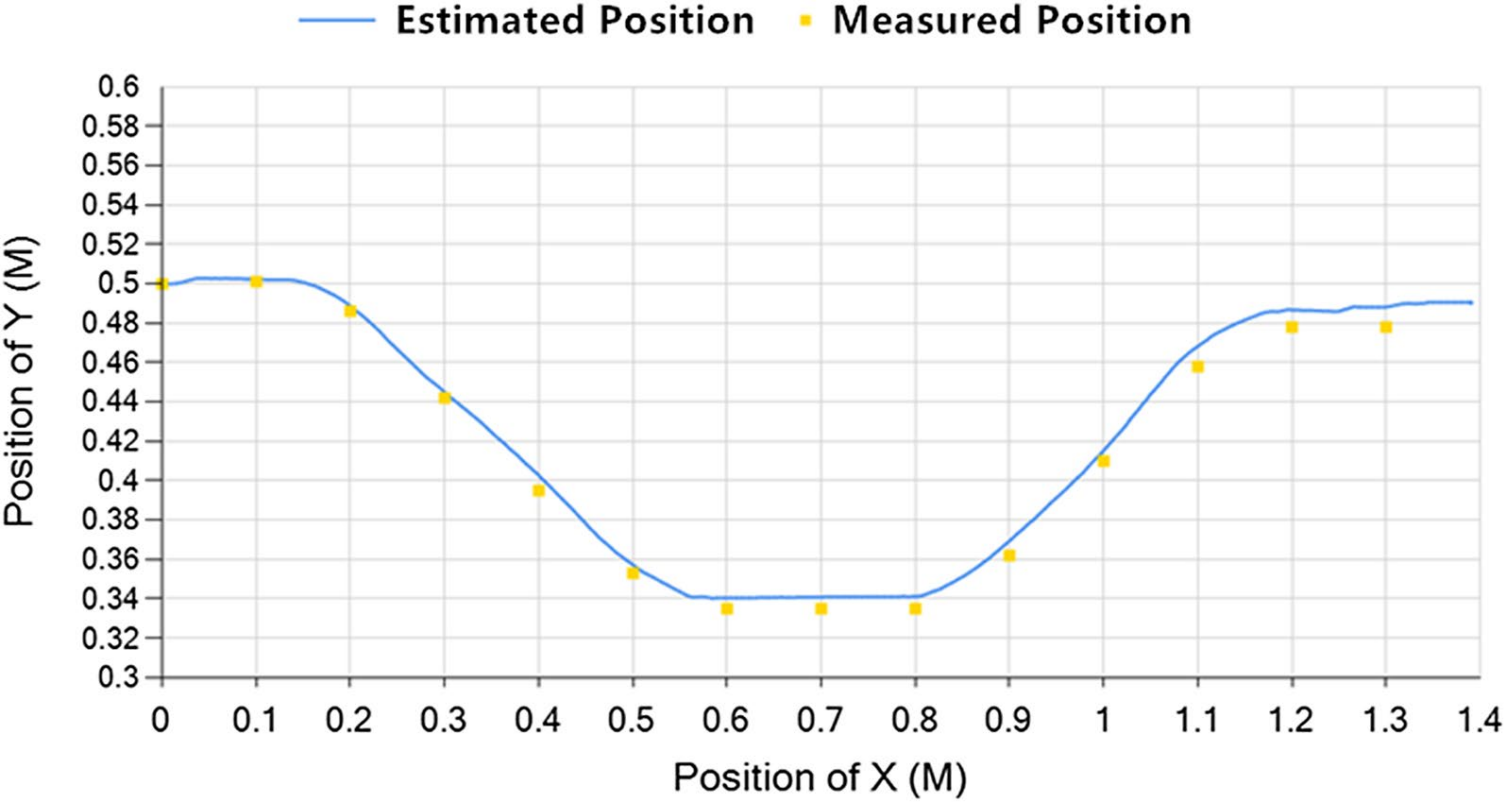
S curve

From Fig. 8, when the vehicle moves in an S curve, it moves as an S curve in the steam generator, and the error would increase when it has a large change of forward angle. But the error is still below 1.1%, it is within the acceptable range. So, the localization is accurate when it moves along S curve in the steam generator.

## Conclusions

Nuclear energy is one of the most important energy on earth. The steam generator secondary side is the key device of a nuclear power station. As an important branch of special robots, the nuclear robot is the most convenient and effective way to inspect the steam generator. This paper describes one robot system with self-localization that could help users in inspecting tubes and locating the robot vehicle inside the steam generator. Experiments are designed to test the robot system. Results show that this system works stably, and the localization is accurate and effective. In future, we will develop some similar industrial robots, such as pipeline inspection robot, to help people complete work conveniently and efficiently.
